# Supplementary material for: Image-based effective feature generation for protein structural class and ligand binding prediction
Source: PeerJ Comput Sci. 2020 Feb 3;6:e253. doi: 10.7717/peerj-cs.253 (PMC7924679; doi:10.7717/peerj-cs.253)
Supplement: Supplemental Information 1 [file peerj-cs-06-253-s001.pdf]

“Image-based effective feature generation for Protein Structural Class and Ligand Binding prediction”

Supplementary File: 01

This supplementary file contains the accuracy table of various SIFT experiments. The explanation of the features is given below.

	Feature	KNN	Naive Bayesian	SVM	Adaboost (J48)	Random Forest
1	SIFT Keypoint Histogram	67.3396	33.0884	42.7546	61.8062	68.2561
2	SIFT Wavelet KeyPoint Histogram	68.8166	22.8681	33.6941	60.0857	71.3228
3	SIFT Keypoint Histogram on QuadTree	63.6202	36.6867	48.8349	60.6027	40.0458
4	SIFT Wavelet Keypoint Histogram on QuadTree	63.6202	36.6867	48.8349	60.6027	69.2664
5	SIFT Canny Keypoint Histogram	66.2992	37.8556	41.0938	51.0679	63.8694
6	SIFT QuadTree descriptor	73.1497	30.6459			74.0835
7	SIFT descriptor 16	72.246	46.178	55.814	64.629	75.09

Table 1: Classifier accuracies for different types of SIFT feature

1. SIFT Keypoint Histogram

It selects how many key points the sift Feature detector has detected according to the x-axis of the image. It sums up the int value of keypoints (which comes as float) on the x-axis.

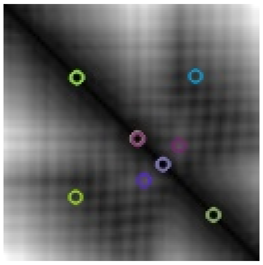

2. **SIFT Wavelet KeyPoint Histogram**

   The image is wavelet transformed and then SIFT Keypoint Histogram is applied.

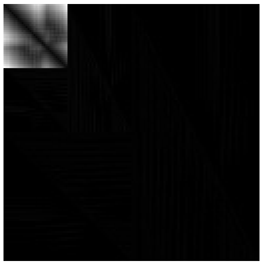

3. **SIFT Keypoint Histogram on QuadTree**

   It splits the image into equally 4 parts and gets histogram of keypoints from the x-axis.

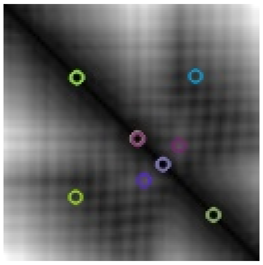

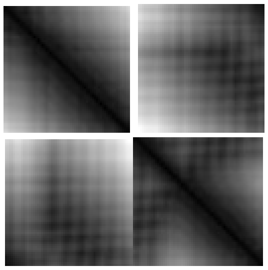

4. **SIFT Wavelet Keypoint Histogram on QuadTree**

   The image is wavelet transformed . and then the previous procedure is done.

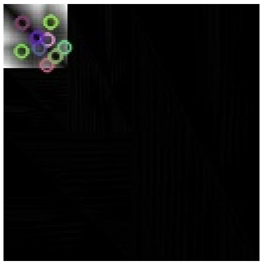

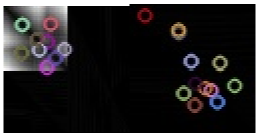

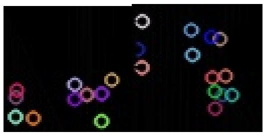

5. **SIFT Canny Keypoint Histogram**

   Canny edge detector is used for transform the images and then SIFT Keypoint Histogram is used.

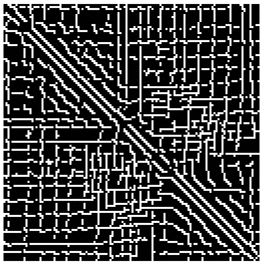

6. **SIFT QuadTree descriptor**

   One descriptor from each of the quad images are taken, so there is 4 descriptor per image as an instance.

7. **SIFT descriptor 16**

   Each image is divided into 16 images and from each image 1 descriptor is taken. So, there are total of 16 descriptors per image.
