# Supplementary material for: Image-based effective feature generation for protein structural class and ligand binding prediction
Source: PeerJ Comput Sci. 2020 Feb 3;6:e253. doi: 10.7717/peerj-cs.253 (PMC7924679; doi:10.7717/peerj-cs.253)
Supplement: Supplemental Information 2 [file peerj-cs-06-253-s002.pdf]

“Image-based effective feature generation for Protein Structural Class and Ligand Binding prediction”

Supplementary File: 02

This supplementary file contains the results of the performance metrics (accuracy, sensitivity, specificity, f1 score) of the feature group ABCDE (Hybrid LBP). The dataset of the feature group is stratified into 10 folds. For each fold, the fold is used as test dataset and the rest of the dataset (9 folds) is used as train dataset. SMOTE is used for each train dataset to balance the classes. Table 1,2,3 and 4 contains the individual result of each stratified dataset and average result for each classifier on the accuracy, sensitivity, specificity, f1 score respectively.

Category	Features	Random Forest	AdaBoost		KNN (5)	Naïve Bayes	SVM
			Random Forest	J48			
Stratified 10-1	HybridLBP	78.6618	78.2098	75.226	50.2712	34.5389	77.2152
Stratified 10-2	HybridLBP	78.5714	78.7523	78.7523	51.2658	33.5443	76.7631
Stratified 10-3	HybridLBP	77.6471	77.8281	76.2896	55.2941	33.5747	76.7421
Stratified 10-4	HybridLBP	77.6471	77.1041	74.9321	50.0452	34.5701	76.2896
Stratified 10-5	HybridLBP	77.3756	76.7421	76.7421	53.2127	33.9367	77.4661
Stratified 10-6	HybridLBP	75.3846	75.9276	76.2896	51.0407	35.2941	78.009
Stratified 10-7	HybridLBP	73.2127	75.2036	75.3846	49.5023	35.8371	76.3801
Stratified 10-8	HybridLBP	75.8371	77.4661	76.1991	49.2308	37.4661	78.371
Stratified 10-9	HybridLBP	76.9231	77.8281	75.7466	52.2172	36.9231	77.0136
Stratified 10-10	HybridLBP	76.3801	77.1041	77.0136	51.6742	37.8281	78.4615
	**Average**	76.764	77.217	76.258	51.375	35.351	77.271

Table 1: Classifier accuracies for each stratified dataset of ABCDE feature group

Category	Features	Random Forest	AdaBoost		KNN (5)	Naïve Bayes	SVM
			Random Forest	J48			
Stratified 10-1	HybridLBP	0.787	0.782	0.752	0.503	0.345	0.772
Stratified 10-2	HybridLBP	0.786	0.788	0.788	0.513	0.335	0.768
Stratified 10-3	HybridLBP	0.776	0.778	0.763	0.553	0.336	0.767
Stratified 10-4	HybridLBP	0.776	0.771	0.749	0.5	0.346	0.763
Stratified 10-5	HybridLBP	0.774	0.767	0.767	0.532	0.339	0.775
Stratified 10-6	HybridLBP	0.754	0.759	0.763	0.51	0.353	0.78
Stratified 10-7	HybridLBP	0.732	0.752	0.754	0.495	0.358	0.764
Stratified 10-8	HybridLBP	0.758	0.775	0.762	0.492	0.375	0.784
Stratified 10-9	HybridLBP	0.769	0.778	0.757	0.522	0.369	0.77
Stratified 10-10	HybridLBP	0.764	0.771	0.77	0.517	0.378	0.785
	**Average**	76.76%	77.21%	76.25%	51.37%	35.34%	77.28%

Table 2: Classifier sensitivity for each stratified dataset of ABCDE feature group

Category	Features	Random Forest	AdaBoost Random Forest	AdaBoost J48	KNN (5)	Naïve Bayes	SVM
Stratified 10-1	HybridLBP	0.938	0.934	0.929	0.927	0.835	0.953
Stratified 10-2	HybridLBP	0.937	0.94	0.937	0.935	0.841	0.952
Stratified 10-3	HybridLBP	0.936	0.934	0.929	0.941	0.833	0.948
Stratified 10-4	HybridLBP	0.939	0.935	0.925	0.925	0.843	0.946
Stratified 10-5	HybridLBP	0.932	0.929	0.93	0.934	0.837	0.951
Stratified 10-6	HybridLBP	0.931	0.93	0.936	0.93	0.841	0.951
Stratified 10-7	HybridLBP	0.924	0.931	0.928	0.925	0.841	0.949
Stratified 10-8	HybridLBP	0.928	0.933	0.925	0.92	0.853	0.952
Stratified 10-9	HybridLBP	0.934	0.937	0.928	0.93	0.841	0.949
Stratified 10-10	HybridLBP	0.935	0.935	0.935	0.926	0.844	0.953
	**Average**	93.34%	93.38%	93.02%	92.93%	84.09%	95.04%

Table 3: Classifier specificity for each stratified dataset of ABCDE feature group

Category	Features	Random Forest	AdaBoost		KNN (5)	Naïve Bayes	SVM
			Random Forest	J48			
Stratified 10-1	HybridLBP	0.785	0.779	0.753	0.53	0.305	0.784
Stratified 10-2	HybridLBP	0.785	0.787	0.788	0.539	0.302	0.779
Stratified 10-3	HybridLBP	0.776	0.776	0.762	0.585	0.304	0.777
Stratified 10-4	HybridLBP	0.779	0.773	0.748	0.521	0.305	0.771
Stratified 10-5	HybridLBP	0.774	0.767	0.766	0.563	0.296	0.786
Stratified 10-6	HybridLBP	0.756	0.761	0.765	0.536	0.324	0.789
Stratified 10-7	HybridLBP	0.733	0.753	0.755	0.524	0.319	0.777
Stratified 10-8	HybridLBP	0.759	0.775	0.758	0.523	0.353	0.792
Stratified 10-9	HybridLBP	0.768	0.778	0.758	0.551	0.337	0.78
Stratified 10-10	HybridLBP	0.762	0.769	0.77	0.549	0.343	0.793
	**Average**	76.77%	77.18%	76.23%	54.21%	31.88%	78.28%

Table 4: Classifier f1 score for each stratified dataset of ABCDE feature group
