# Supplementary material for: Image-based effective feature generation for protein structural class and ligand binding prediction
Source: PeerJ Comput Sci. 2020 Feb 3;6:e253. doi: 10.7717/peerj-cs.253 (PMC7924679; doi:10.7717/peerj-cs.253)
Supplement: Supplemental Information 3 [file peerj-cs-06-253-s003.pdf]

“Image-based effective feature generation for Protein Structural Class and Ligand Binding prediction”

Supplementary File: 03

This supplementary file contains the results of the performance metrics (accuracy, sensitivity, specificity, f1 score) of the feature group ComogPhog. The dataset of the feature group is stratified into 10 folds. For each fold, the fold is used as test dataset and the rest of the dataset (9 folds) is used as train dataset. SMOTE is used for each train dataset to balance the classes. Table 1,2,3 and 4 contains the individual result of each stratified dataset and average result for each classifier on the accuracy, sensitivity, specificity, f1 score respectively.

Category	Features	Random Forest	AdaBoost		KNN (5)	Naïve Bayes	SVM
			Random Forest	J48			
Stratified 10-1	ComogPHOG	73.8698	74.141	72.604	69.6203	56.6908	76.7631
Stratified 10-2	ComogPHOG	71.6998	72.1519	71.2477	67.2694	55.1537	76.4014
Stratified 10-3	ComogPHOG	73.2127	72.8507	72.2172	68.0543	54.8416	75.9276
Stratified 10-4	ComogPHOG	75.4751	75.2941	71.6742	67.5113	58.4615	75.4751
Stratified 10-5	ComogPHOG	75.3846	75.0226	73.5747	68.9593	55.8371	77.1946
Stratified 10-6	ComogPHOG	77.1041	78.19	75.2036	69.5928	58.009	77.9186
Stratified 10-7	ComogPHOG	73.3032	74.0271	72.0362	63.5294	57.2851	74.0271
Stratified 10-8	ComogPHOG	73.1222	73.8462	71.6742	70.6787	56.3801	78.371
Stratified 10-9	ComogPHOG	75.5656	75.8371	72.6697	67.5113	55.9276	76.3801
Stratified 10-10	ComogPHOG	74.0271	74.1176	72.1267	67.6018	55.4751	76.8326
	**Average**	74.276	74.548	72.503	68.033	56.406	76.529

Table 1: Classifier accuracies for each stratified dataset of ComogPHOG

Category	Features	Random Forest	AdaBoost		KNN (5)	Naïve Bayes	SVM
			Random Forest	J48			
Stratified 10-1	ComogPHOG	0.739	0.741	0.726	0.696	0.567	0.768
Stratified 10-2	ComogPHOG	0.717	0.722	0.712	0.673	0.552	0.764
Stratified 10-3	ComogPHOG	0.732	0.729	0.722	0.681	0.548	0.759
Stratified 10-4	ComogPHOG	0.755	0.753	0.717	0.675	0.585	0.755
Stratified 10-5	ComogPHOG	0.754	0.75	0.736	0.69	0.558	0.772
Stratified 10-6	ComogPHOG	0.771	0.782	0.752	0.696	0.58	0.779
Stratified 10-7	ComogPHOG	0.733	0.74	0.72	0.635	0.573	0.74
Stratified 10-8	ComogPHOG	0.731	0.738	0.717	0.707	0.564	0.784
Stratified 10-9	ComogPHOG	0.756	0.758	0.727	0.675	0.559	0.764
Stratified 10-10	ComogPHOG	0.74	0.741	0.721	0.676	0.555	0.768
	**Average**	74.28%	74.54%	72.50%	68.04%	56.41%	76.53%

Table 2: Classifier sensitivity for each stratified dataset of ComogPHOG

Category	Features	Random Forest	AdaBoost Random Forest	AdaBoost J48	KNN (5)	Naïve Bayes	SVM
Stratified 10-1	ComogPHOG	0.931	0.934	0.929	0.931	0.893	0.943
Stratified 10-2	ComogPHOG	0.93	0.93	0.928	0.934	0.887	0.945
Stratified 10-3	ComogPHOG	0.931	0.928	0.924	0.929	0.885	0.945
Stratified 10-4	ComogPHOG	0.937	0.936	0.925	0.928	0.897	0.947
Stratified 10-5	ComogPHOG	0.935	0.936	0.932	0.934	0.895	0.947
Stratified 10-6	ComogPHOG	0.946	0.948	0.935	0.933	0.898	0.949
Stratified 10-7	ComogPHOG	0.93	0.935	0.929	0.916	0.913	0.941
Stratified 10-8	ComogPHOG	0.932	0.934	0.925	0.931	0.893	0.951
Stratified 10-9	ComogPHOG	0.939	0.941	0.927	0.938	0.895	0.947
Stratified 10-10	ComogPHOG	0.93	0.931	0.924	0.924	0.893	0.949
	**Average**	93.41%	93.53%	92.78%	92.98%	89.49%	94.64%

Table 3: Classifier specificity for each stratified dataset of ComogPHOG

Category	Features	Random Forest	AdaBoost		KNN (5)	Naïve Bayes	SVM
			Random Forest	J48			
Stratified 10-1	ComogPHOG	0.739	0.743	0.728	0.704	0.565	0.774
Stratified 10-2	ComogPHOG	0.719	0.723	0.714	0.686	0.548	0.769
Stratified 10-3	ComogPHOG	0.734	0.731	0.725	0.69	0.544	0.769
Stratified 10-4	ComogPHOG	0.757	0.752	0.719	0.687	0.586	0.764
Stratified 10-5	ComogPHOG	0.755	0.752	0.738	0.702	0.559	0.781
Stratified 10-6	ComogPHOG	0.774	0.784	0.754	0.709	0.585	0.786
Stratified 10-7	ComogPHOG	0.733	0.74	0.723	0.646	0.592	0.749
Stratified 10-8	ComogPHOG	0.731	0.74	0.718	0.714	0.567	0.792
Stratified 10-9	ComogPHOG	0.755	0.76	0.727	0.694	0.564	0.77
Stratified 10-10	ComogPHOG	0.739	0.741	0.721	0.683	0.553	0.774
	**Average**	74.36%	74.66%	72.67%	69.15%	56.63%	77.28%

Table 4: Classifier f1 score for each stratified dataset of ComogPHOG
