# Supplementary material for: Image-based effective feature generation for protein structural class and ligand binding prediction
Source: PeerJ Comput Sci. 2020 Feb 3;6:e253. doi: 10.7717/peerj-cs.253 (PMC7924679; doi:10.7717/peerj-cs.253)
Supplement: Supplemental Information 4 [file peerj-cs-06-253-s004.pdf]

“Image-based effective feature generation for Protein Structural Class and Ligand Binding prediction”

Supplementary File: 04

This supplementary file contains the comparison of the performance metrics (accuracy, sensitivity, specificity, f1 score) between the feature group ABCDE (Hybrid LBP) and ComogPHOG.

Performance Metric	Features	Random Forest	AdaBoost Random Forest	AdaBoost J48	KNN (5)	Naïve Bayes	SVM
Accuracy	HybridLBP	**76.76406**	**77.21659**	**76.25756**	51.37542	35.35132	**77.27113**
	ComogPHOG	74.27642	74.54783	72.50282	**68.03286**	**56.40616**	76.52912
Sensitivity	HybridLBP	**0.7676**	**0.7721**	**0.7625**	0.5137	0.3534	**0.7728**
	ComogPHOG	0.7428	0.7454	0.725	**0.6804**	**0.5641**	0.7653
Specificity	HybridLBP	0.9334	0.9338	**0.9302**	0.9293	0.8409	**0.9504**
	ComogPHOG	**0.9341**	**0.9353**	0.9278	**0.9298**	**0.8949**	0.9464
F1 Score	HybridLBP	**0.7677**	**0.7718**	**0.7623**	0.5421	0.3188	**0.7828**
	ComogPHOG	0.7436	0.7466	0.7267	**0.6915**	**0.5663**	0.7728
