# Supplementary material for: Image-based effective feature generation for protein structural class and ligand binding prediction
Source: PeerJ Comput Sci. 2020 Feb 3;6:e253. doi: 10.7717/peerj-cs.253 (PMC7924679; doi:10.7717/peerj-cs.253)
Supplement: Supplemental Information 5 [file peerj-cs-06-253-s005.pdf]

“Image-based effective feature generation for Protein Structural Class and Ligand Binding prediction”

Supplementary File: 05

This supplementary file contains the comparison of the performance metrics (accuracy, sensitivity, specificity, f1 score) between our Similarity-Based Clustering algorithm and the existing ML algorithms based on the dataset including outlier and inlier negative data.

Features	AdaBoost (J48)	KNN (1)	KNN (5)	Random Forest	SVM	Naïve Bayesian	Our Method (5)	Our Method (3)
**HybridLBP (inlier)**	99.70%	70.71%	84.50%	99.81%	99.53%	95.20%	48.47%	49.07%
**HybridLBP (outlier)**	99.68%	73.44%	83.99%	99.66%	99.36%	73.44%	57.95%	58.12%
**ComogPHOG (inlier)**	99.75%	76.09%	78.26%	99.81%	99.28%	64.54%	45.62%	46.51%
**ComogPHOG (outlier)**	98.01%	94.83%	92.92%	97.83%	97.22%	79.27%	69.83%	68.67%

Table 1 Comparison of Accuracy

Features	AdaBoost (J48)	KNN (1)	KNN (5)	Random Forest	SVM	Naïve Bayesian	Our Method (5)	Our Method (3)
**HybridLBP (inlier)**	99.50%	56.50%	69.20%	99.60%	99.10%	90.40%	55.51%	57.08%
**HybridLBP (outlier)**	99.60%	57.90%	68.30%	99.60%	98.90%	56.10%	75.82%	77.90%
**ComogPHOG (inlier)**	99.50%	65.60%	60.10%	99.60%	98.60%	42.50%	39.91%	38.51%
**ComogPHOG (outlier)**	97.10%	91.50%	87.40%	98.30%	95.80%	64.60%	63.71%	62.52%

Table 2 Comparison of Sensitivity

Features	AdaBoost (J48)	KNN (1)	KNN (5)	Random Forest	SVM	Naïve Bayesian	Our Method (5)	Our Method (3)
**HybridLBP (inlier)**	99.90%	84.90%	99.80%	100.00%	100.00%	100.00%	41.49%	41.11%
**HybridLBP (outlier)**	99.80%	88.90%	99.70%	99.70%	99.80%	90.80%	40.09%	38.34%
**ComogPHOG (inlier)**	100.00%	86.60%	96.40%	100.00%	100.00%	86.60%	51.32%	54.50%
**ComogPHOG (outlier)**	98.90%	98.20%	98.40%	97.30%	98.60%	93.90%	75.92%	74.79%

Table 3 Comparison of Specificity

Features	AdaBoost (J48)	KNN (1)	KNN (5)	Random Forest	SVM	Naïve Bayesian	Our Method (5)	Our Method (3)
**HybridLBP (inlier)**	99.70%	65.90%	81.70%	99.80%	99.50%	95.00%	51.83%	52.84%
**HybridLBP (outlier)**	99.70%	68.60%	81.00%	99.70%	99.40%	67.90%	64.32%	65.01%
**ComogPHOG (inlier)**	99.70%	73.30%	73.40%	99.80%	99.30%	54.50%	42.30%	41.87%
**ComogPHOG (outlier)**	98.00%	94.60%	92.50%	97.80%	97.20%	75.70%	67.84%	66.60%

Table 4 Comparison of F1 Score

For creating negative data for the Ligand-Binding dataset, we've used Local Outlier Factor from scikit-learn library to detect if an unseen Protein-Ligand pair falls in the same region where the positive data are condensed into. If it is in the region, it is inlier, else it is marked as an outlier. This method is called novelty detection. Firstly, we've taken random inliers as negative training data. But somehow most of the algorithms overfit. Similarly, taking random outliers as negative data was giving the same overfitting complication. As you can see from the above tables, most of the MLs are overfitted. The negative instances (inlier/outlier) were taken in a pattern that good MLs can find that pattern and easily detect class values thus giving high-performance scores. This is causing the overfitting problem. We can't be sure if these negative data (outliers/inliers) are close to actual negative data as we don't have them. This is why we didn't use these features. So, random undersampling and clustering-based under sampling are more reliable than outlier detection as these methods don't give overfitting issues.
