# Supplementary material for: Image-based effective feature generation for protein structural class and ligand binding prediction
Source: PeerJ Comput Sci. 2020 Feb 3;6:e253. doi: 10.7717/peerj-cs.253 (PMC7924679; doi:10.7717/peerj-cs.253)
Supplement: Supplemental Information 6 [file peerj-cs-06-253-s006.pdf]

“Image-based effective feature generation for Protein Structural Class and Ligand Binding prediction”

Supplementary File: 06

This supplementary file contains the comparison of the performance metrics (accuracy, sensitivity, specificity, f1 score) between our Similarity-Based Clustering algorithm and the existing ML algorithms.

Features	AdaBoost (J48)	KNN (1)	KNN (5)	Random Forest	SVM	Naïve Bayesian	Our Method (5)	Our Method (3)
HybridLBP (random)	41.54%	35.04%	41.74%	20.05%	36.35%	46.36%	53.39%	54.16%
HybridLBP (cluster)	54.36%	45.08%	53.37%	57.75%	53.93%	51.53%	53.66%	53.98%
ComogPHOG (random)	49.17%	44.56%	47.54%	14.56%	34.26%	47.61%	54.53%	55.29%

Table 1 Comparison of Accuracy

Features	AdaBoost (J48)	KNN (1)	KNN (5)	Random Forest	SVM	Naïve Bayesian	Our Method (5)	Our Method (3)
HybridLBP (random)	41.90%	29.80%	36.30%	21.90%	33.70%	43.10%	64.44%	66.21%
HybridLBP (cluster)	52.90%	39.40%	48.70%	53.40%	51.00%	37.60%	64.13%	65.82%
ComogPHOG (random)	95.20%	44.70%	47.60%	16.10%	29.70%	11.30%	52.90%	50.68%

Table 2 Comparison of Sensitivity

Features	AdaBoost (J48)	KNN (1)	KNN (5)	Random Forest	SVM	Naïve Bayesian	Our Method (5)	Our Method (3)
HybridLBP (random)	41.20%	40.30%	47.20%	18.20%	39.00%	49.60%	42.38%	42.19%
HybridLBP (cluster)	55.80%	50.80%	58.00%	62.20%	56.90%	65.50%	43.21%	42.19%
ComogPHOG (random)	3.10%	44.50%	47.50%	13.00%	38.80%	83.90%	56.13%	59.85%

Table 3 Comparison of Specificity

Features	AdaBoost (J48)	KNN (1)	KNN (5)	Random Forest	SVM	Naïve Bayesian	Our Method (5)	Our Method (3)
**HybridLBP (random)**	41.80%	34.90%	38.40%	21.50%	34.60%	44.60%	58.01%	59.06%
**HybridLBP (cluster)**	53.70%	41.80%	51.10%	55.80%	52.50%	43.70%	58.03%	58.83%
**ComogPHOG (random)**	65.20%	44.60%	47.60%	15.80%	31.10%	17.80%	53.75%	53.11%

Table 4 Comparison of F1 Score

Selection of performance metric:

In terms of Protein-Ligand Binding Prediction, we didn't have negative instances. So, we had to generate them by two different methods. So, all of the negative instances are actually artificial data. That is a clear indication that judging the algorithms based on the accuracy score is not valid as the scores are influenced by the artificial negative instances. Sensitivity is the true positive rate which is the percentage of positively classified instances among the actual positive instances. On the other hand, Precision is the percentage of positive instances among the positively classified instances. While both are scores based on positive data, the sensitivity score is more logical than the precision score as it is based on predicted positive instances whereas sensitivity is based on actual positive instances of the train set. Furthermore, the F1 score is the harmonic mean of sensitivity and precision, which makes it another valid performance metric for judging the algorithms as the Sensitivity score. That's why sensitivity and F1 score are shown in the paper.

Correlation between Sensitivity & Specificity:

Sometimes higher sensitivity can be offset by low specificity. But here, this is not the case. Both sensitivity and specificities of our algorithm are better than other machine learning algorithms and they are not counterbalancing each other. Overall scores on these 3 different datasets are not as good as researchers desire to get. This happend due to the absence of negative data. Inspite of this impurity in data, our algorithm outperforms other ML algorithms.

Discussion:

You've probably noticed the high sensitivity in ComogPHOG (random) dataset. This is actually an overfitting problem. Because this dataset has very low specificity which is the true negative rate. This is the actual case of higher sensitivity which is an offset by low specificity.
