# Supplementary material for: Image-based effective feature generation for protein structural class and ligand binding prediction
Source: PeerJ Comput Sci. 2020 Feb 3;6:e253. doi: 10.7717/peerj-cs.253 (PMC7924679; doi:10.7717/peerj-cs.253)
Supplement: Supplemental Information 7 [file peerj-cs-06-253-s007.pdf]

“Image-based effective feature generation for Protein Structural Class and Ligand Binding prediction”

Supplementary File: 07

This supplementary file contains the results of the performance metrics (accuracy, sensitivity, specificity, f1 score) of the different feature proposed on our paper. The dataset of the feature group is stratified into 10 folds. For each fold, the fold is used as test dataset and the rest of the dataset (9 folds) is used as train dataset. SMOTE is used for each train dataset to balance the classes. Table 1,2,3 and 4 contains the individual result of each stratified dataset and average result for each classifier on the accuracy, sensitivity, specificity, f1 score respectively.

Image Type	Features	Random Forest	Adaboost		KNN (5)	Naïve Bayes	SVM
			Random Forest	J48			
scaled	A	75.1356	75.0452	68.6257	50.8137	50.6329	69.1682
non-scaled	A	75.859	75.3165	72.1519	53.8879	34.5389	68.4448
scaled	B	30.8318	31.5552	24.8644	21.9711	8.5895	17.4503
non-scaled	B	42.8571	41.953	37.2514	30.8318	25.4069	23.2369
none	C	39.5118	37.8843	36.0759	15.8228	5.6058	25.7685
scaled	D	74.774	74.774	70.9765	**69.4394**	48.0108	67.6311
scaled	E	74.0506	73.9602	72.604	61.302	**56.2387**	64.7378
	AB	73.5081	73.5081	71.3382	51.7179	32.4593	68.8969
	ABC	74.2315	73.8698	69.8011	27.3056	32.2785	68.6257
	ABCD	77.8481	77.2152	72.8752	41.7722	33.3635	74.6835
	ABCDE	**78.6618**	**78.2098**	**75.226**	50.2712	34.5389	**77.2152**

Table 1: Classifier accuracies for different feature group

Image Type	Features	Random Forest	Adaboost		KNN (5)	Naïve Bayes	SVM
			Random Forest	J48			
scaled	A	0.751	0.75	0.686	0.508	0.506	0.692
non-scaled	A	0.759	0.753	0.722	0.539	0.345	0.684
scaled	B	0.308	0.316	0.249	0.22	0.086	0.175
non-scaled	B	0.429	0.42	0.373	0.308	0.254	0.232
none	C	0.395	0.379	0.361	0.158	0.056	0.258
scaled	D	0.748	0.748	0.71	0.694	0.48	0.676
scaled	E	0.741	0.74	0.726	0.613	0.562	0.647
	AB	0.735	0.735	0.713	0.517	0.325	0.689
	ABC	0.742	0.739	0.698	0.273	0.323	0.686
	ABCD	0.778	0.772	0.729	0.418	0.334	0.747
	ABCDE	0.787	0.782	0.752	0.503	0.345	0.772

Table 2: Classifier sensitivity for different feature group

Image Type	Features	Random Forest	Adaboost		KNN (5)	Naïve Bayes	SVM
			Random Forest	J48			
scaled	A	0.926	0.929	0.908	0.933	0.887	0.942
non-scaled	A	0.929	0.929	0.92	0.932	0.826	0.943
scaled	B	0.785	0.788	0.787	0.833	0.935	0.871
non-scaled	B	0.828	0.827	0.815	0.858	0.841	0.86
none	C	0.79	0.782	0.797	0.889	0.971	0.868
scaled	D	0.94	0.939	0.924	0.939	0.905	0.947
scaled	E	0.935	0.936	0.928	0.938	0.907	0.951
	AB	0.921	0.922	0.915	0.907	0.83	0.941
	ABC	0.919	0.919	0.906	0.899	0.827	0.935
	ABCD	0.933	0.933	0.92	0.912	0.83	0.948
	ABCDE	0.938	0.934	0.929	0.927	0.835	0.953

Table 3: Classifier specificity for different feature group

Image Type	Features	Random Forest	Adaboost		KNN (5)	Naïve Bayes	SVM
			Random Forest	J48			
scaled	A	0.749	0.75	0.69	0.534	0.514	0.711
non-scaled	A	0.758	0.754	0.723	0.561	0.297	0.698
scaled	B	0.29	0.297	0.25	0.235	0.093	0.193
non-scaled	B	0.405	0.399	0.363	0.31	0.183	0.193
none	C	0.353	0.336	0.35	0.167	0.025	0.268
scaled	D	0.756	0.756	0.714	0.708	0.513	0.703
scaled	E	0.745	0.743	0.73	0.641	0.586	0.685
	AB	0.735	0.735	0.716	0.524	0.275	0.7
	ABC	0.741	0.737	0.696	0.271	0.272	0.697
	ABCD	0.777	0.771	0.73	0.425	0.286	0.759
	ABCDE	0.785	0.779	0.753	0.53	0.305	0.784

Table 4: Classifier f1 score for different feature group
